# Quenched pinning and collective dislocation dynamics

**DOI:** 10.1038/srep10580

**Published:** 2015-05-29

**Authors:** Markus Ovaska, Lasse Laurson, Mikko J. Alava

**Affiliations:** 1 COMP Centre of Excellence, Department of Applied Physics, Aalto University, P.O. Box 11100, 00076 Aalto, Espoo, Finland

## Abstract

Several experiments show that crystalline solids deform in a bursty and intermittent fashion. Power-law distributed strain bursts in compression experiments of micron-sized samples, and acoustic emission energies from larger-scale specimens, are the key signatures of the underlying critical-like collective dislocation dynamics - a phenomenon that has also been seen in discrete dislocation dynamics (DDD) simulations. Here we show, by performing large-scale two-dimensional DDD simulations, that the character of the dislocation avalanche dynamics changes upon addition of sufficiently strong randomly distributed quenched pinning centres, present e.g. in many alloys as immobile solute atoms. For intermediate pinning strength, our results adhere to the scaling picture of depinning transitions, in contrast to pure systems where dislocation jamming dominates the avalanche dynamics. Still stronger disorder quenches the critical behaviour entirely.

The origin of crackling noise^1^ in crystal plasticity has an appealing explanation in terms of a non-equilibrium phase transition^2^,^3^,^4^: If the externally applied stress is high enough, a sample is in a regime of continuous flow or yielding, while at small stress values and low temperatures one would expect the yielding activity to stop after a transient. The existence of a “yielding transition” separating these two regimes, envisaged to take place at a critical value of the applied stress, and corresponding to a vanishing plastic deformation rate, would then give rise to a natural explanation for the observed scale-free avalanche dynamics^5^,^6^,^7^,^8^,^9^. Such a picture has been used in analogy to other systems, including the depinning transition of domain walls in disordered ferromagnets, underlying the magnetic field driven jerky domain walls motion, giving rise to the Barkhausen effect^10^,^11^,^12^.

The irreversible deformation process of crystalline solids is a consequence of the stress-driven motion of dislocations, line-like defects of the crystal lattice, which interact with each other via their anisotropic long-range stress fields. Due to these interactions, in combination with constraints due to the underlying crystal structure on their motion, dislocations tend to form various complicated metastable structures. Thus, the term “dislocation jamming”^13^,^14^,^15^ has been coined, to describe the tendency of dislocations to get stuck due to many-body dislocation interactions. This mechanism is then expected to be behind the emergence of a finite yield stress in “pure” crystals, without a significant population of additional defects, such as solute atoms, or the complications of e.g. grain boundaries. The character of the dislocation jamming transition in such “pure” DDD models has been analysed from various angles^13^,^14^,^16^,^17^,^18^,^19^. A recent study found^19^ that the scaling exhibited by the strain bursts within a two-dimensional (2*d*) pure DDD model is fundamentally different from that expected within the mean field, or high-dimensional limit of the pinning/depinning scenario^20^, often assumed to describe bursty plastic deformation^21^: 2*d* dislocation dynamics seems to exhibit critical signatures with “anomalous” properties not only in the proximity of the yielding transition, but also at very low external stresses. A detailed study is so far missing in three dimensions, though 3*d* DDD simulations of dislocation avalanches seem to reproduce partially the typical single crystal compression results^7^, and to be close to the mean-field case.

In reality, plastic deformation or dislocation glide is usually further complicated by the presence of various kinds of defects - precipitates, grain boundaries, vacancies, and solute atoms - that interact with the dislocations, and thus interfere with the deformation process^22^,^23^; indeed, much of metallurgy is based on the practical utilisation of this phenomenon, to optimise hardness or ductility^24^. An analogous, well-known system is given by high-temperature superconductors with vortex pinning^25^. Here, we study the effect of disorder on collective dislocation dynamics. To this end, we generalise the standard, two-dimensional (2*d*) DDD models^26^,^27^ to include a random arrangement of *N*_s_ quenched pinning centres. This random pinning landscape could be due to e.g. solute atoms with a low mobility; however, the detailed nature of the pinning centres is irrelevant for our conclusions, and in general one expects on the basis of the theory of depinning of elastic manifolds in random media that the microscopic details are not important^11^,^20^. A snapshot of the 2*d* system with pinning centres interacting with the dislocations^28^ is shown in Fig. 1.

The disorder immediately widens the phase diagram of the DDD models at a constant applied stress so that the (candidate) order parameter, shear rate, becomes a function of both the applied shear stress and the relative strengths of the pinning and the dislocation-dislocation interactions. For strong enough disorder, we find a yielding transition that agrees with the standard depinning scaling scenario, exhibiting the typical signatures of power-law distributions for both avalanche sizes and durations, with the cut-offs of the distributions displaying power-law divergences at a critical applied stress: yielding becomes depinning, and jamming becomes pinning. The set of critical exponents characterising these distributions is found to be different from that of mean field depinning. With still stronger disorder, the collective behaviour disappears, as is indicated by the qualitative phase diagram in the inset of Fig. 1. A special point of the phase diagram corresponding to the low-disorder limit is given by the corresponding pure system^19^, where the scaling behaviour is completely different from those of the above-mentioned two phases where pinning plays a role. We next explore these two novel phases, in particular by considering the scaling properties of dislocation avalanches.

## Results

**Figure 1:**
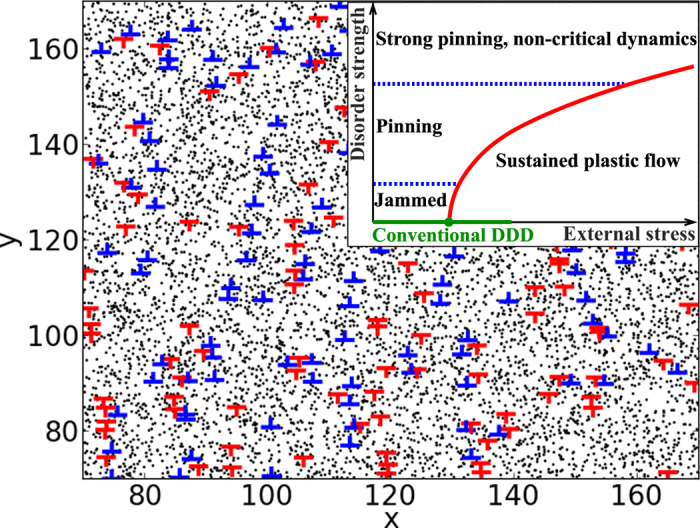
A snapshot of a two-dimensional dislocation assembly with quenched pinning centres, and a phase diagram portraying the nature of dislocation dynamics . The main figure shows a part of the system: Edge dislocations with positive and negative Burgers vectors are shown in blue and red, respectively, and black dots denote randomly positioned quenched pinning sites (solute atoms). The total system size is *L* = 200*b* and it contains *N*_d_ ≈ 900 dislocations and *N*_s_ = 32000 pinning centres. The inset shows a schematic phase diagram in the space spanned by external stress and strength of the quenched disorder. As the disorder strength increases for a fixed external stress, jamming becomes pinning, and finally critical dynamics ceases at very strong disorder.

In order to address and characterise the various aspects of collective dislocation dynamics under the influence of disorder, we consider an extension of the standard 2*d* DDD model^26^,^27^ with single-slip geometry, with the additional ingredient of a quenched pinning field, see Fig. 1 and Methods for details. The model consists of *N*_d_ edge dislocations with an equal number of positive and negative Burgers vectors (with the blue and red symbols in Fig. 1 corresponding to the signs of the Burgers vectors, *s*_*n*_ = +1 and −1, respectively), interacting via their long-range anisotropic stress fields, and gliding along the *x* direction within a square simulation box of linear size *L*. The quenched pinning field is modelled by including *N*_s_ randomly distributed immobile pinning centres, or solute atoms (shown as black dots in Fig. 1), interacting with the dislocations with an interaction strength *A*. Overdamped dynamics is assumed, such that the velocity *v*_*n*_ of the *n*th dislocation is proportional to the total stress (with contributions from interactions with other dislocations, pinning centres and the external stress) acting on it. While not including all the details of full three-dimensional systems with flexible dislocation lines^7^, the model is expected to capture the essential features of the crossover from jamming (dislocations getting stuck to each other) to pinning (dislocations getting stuck to quenched pinning centres), and is simple enough to allow collecting high quality statistics in numerical simulations, an advantage of the 2*d* system over the 3*d* ones. We have verified that our results presented below are robust with respect to changes in details of the pinning potential - as expected on the account of the analogy with depinning models - and are free of any clear finite-size effects detrimental to our results (see Supplementary Figs. S1 and S2). Following the standard procedure of DDD simulations^13^, initially random arrangements of dislocations are first let to relax in zero external stress, *σ*_ext_ = 0, to reach metastable arrangements. Then, the external stress is switched on, and the time-evolution of the system is monitored.

### Depinning transition of the dislocation ensemble

**Figure 2:**
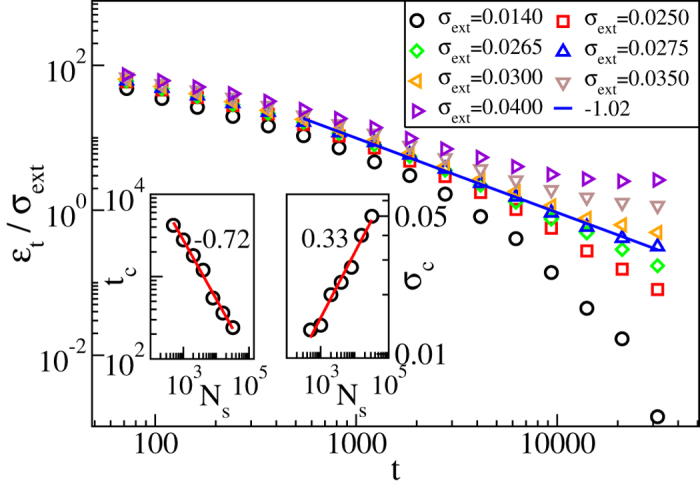
Relaxation of the order parameter exhibits typical power-law behaviour of depinning transitions. Main figure shows the time dependence of the strain rate *ε*_*t*_ for different applied stress values, in a system with *N*_s_ = 8000 pinning sites or solute atoms, of strength *A* = 0.05. For *σ*_ext_ close to *σ*_c_ = 0.0275, *ε*_*t*_ decays as a power law of time, *ε*_*t*_ ∝ *t*^−*θ*^, with *θ* ≈ 1.0. The insets show the dependence of the initial transient time *t*_c_ after which the *t*^−1^ power law decay sets in (left) and the corresponding critical stress *σ*_c_ (right) on the number of solutes *N*_s_, while *A* is kept constant.

Dislocation dynamics in a disordered background is expected to be complicated, with transients and relaxations typical of glassy systems, as is the case also for the depinning of elastic manifolds in random media. The first issue we explore is the response of the system to a constant external stress *σ*_ext_ (“constant control parameter”) at zero temperature. In the steady state, the number of dislocations is *N*_d _≈ 800–900 within a rectangular system of linear size *L* = 200*b* (with *b* the magnitude of the Burgers vector of the dislocations). To tune the pinning strength, we vary the number of pinning centres/solutes in the range *N*_s_ = 500–32000, and set *A* = 0.05. Fig. 2 shows that the time-dependent strain rate 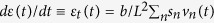 (the “order parameter”) decays exponentially to zero for small *σ*_ext_, while for larger *σ*_ext_ a crossover to a steady state with a non-zero *σ*_ext_-dependent *ε*_*t*_ can be observed. For an intermediate, critical value *σ*_ext_ = *σ*_c_, *ε*_*t*_ decays as a power law of time, *ε*_*t*_ ∝ *t*^−*θ*^, with *θ* ≈ 1.0. Thus, quenched disorder changes the large-scale dynamics of the system, as for a pure system one obtains the well-known 2*d* Andrade law exponent *θ* ≈ 2/3^13^. The *θ*-exponent has a value close to the mean-field depinning one (unity), but a glance at the insets of Fig. 2 reveals that the collective dislocation dynamics is not as simple as that would suggest. The cross-over time *t*_c_ to the power-law relaxation regime decreases with increasing number *N*_s_ of pinning sites, and the critical stress *σ*_c_ obviously increases with *N*_s_. Important is, however, that both the scalings are power-law -like. The behaviour of *σ*_c_(*N*_s_) implies that the dislocations sample the quenched landscape collectively: the power-law relation is not linear in *N*_s_ but scales with an exponent smaller than unity, close to 1/3. This is typical of random manifolds, as is seen from Larkin length arguments in many cases^23^.

### Dislocation avalanches

Then we proceed to study deformation avalanches as a typical, experimental signature of criticality. Similarly to micro-pillar compression experiments^5^,^6^, or to recent numerical studies of the pure DDD model^19^, we apply an adiabatic stress-ramp protocol such that individual, consecutive avalanches can be identified and analysed. We can thus follow the evolution of the deformation bursts all the way up to the yield stress. Starting, as before, from a relaxed configuration, *σ*_ext_ is increased at a slow constant rate *σ*_ext,*t*_ (we consider *σ*_ext,*t*_-values ranging from 2.5 × 10^−7^ to 2.5 × 10^−6^), until the collective dislocation velocity 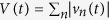
increases above a small threshold value *V*_th_ = 10^−4^. We define an avalanche as a continuous occurrence of *V*(*t*) > *V*_th_, and keep *σ*_ext_ constant until *V*(*t*) falls again below *V*_th_. The total strain increment 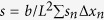 accumulated during such an avalanche is taken to be the avalanche size *s*, and we also consider the statistics of the avalanche durations *T*. Once *V*(*t*) < *V*_th_, and the avalanche has finished, the stress is again ramped up at a rate *σ*_ext,*t*_, until the next avalanche is triggered.

**Figure 3:**
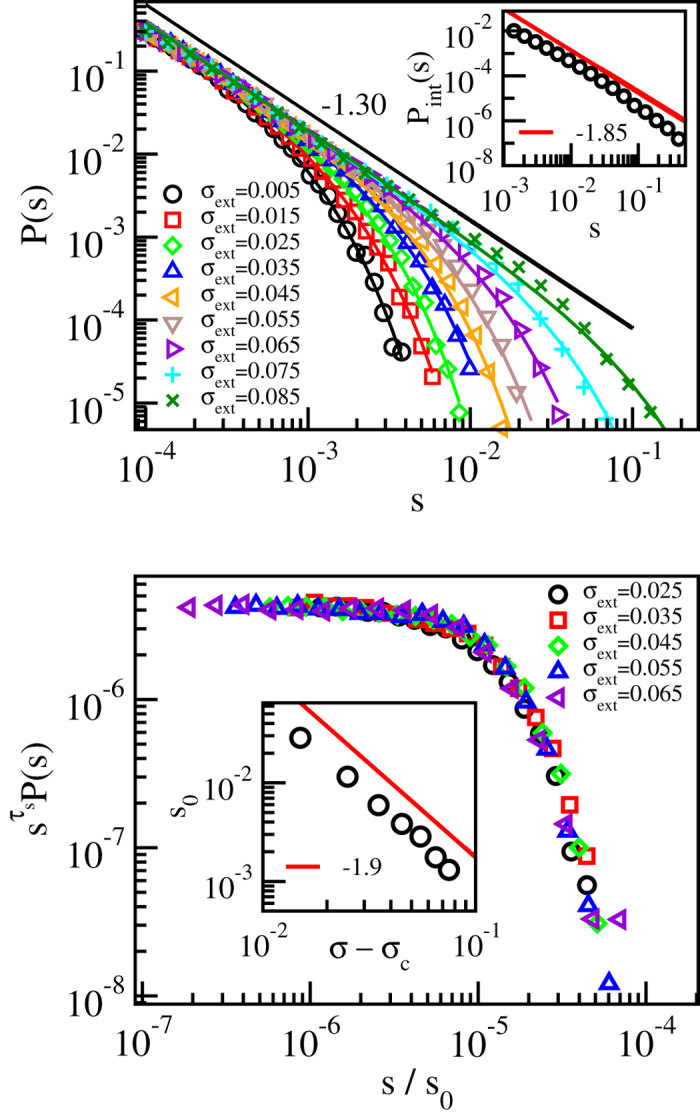
Critical scaling of the strain burst size distributions is depinning-like. (**a**) Distributions *P*(*s*) of the slip avalanche sizes *s* for various stress bins below the critical stress *σ*_c_ ≈ 0.09, in a system with *N*_s_ = 32000 and *A* = 0.1, showing that the *τ*_*s*_-exponent has a value close to 1.30. The solid lines correspond to fits of equation (1) with *f*(*x*) = exp(−*x*) to the data. The inset shows the corresponding stress-integrated distribution, with *τ*_*s*,int_ ≈ 1.85. (**b**) A data collapse of the *P*(*s*) distributions, with *τ*_*s*_ = 1.3 and 1/*σ* = 1.9. The inset shows the cutoff avalanche size *s*_0_ obtained from the fits shown in the top panel as a function of *σ*_c_ − *σ*_ext_, confirming the value of 1/*σ* = 1.9 used in the data collapse.

**Figure 4:**
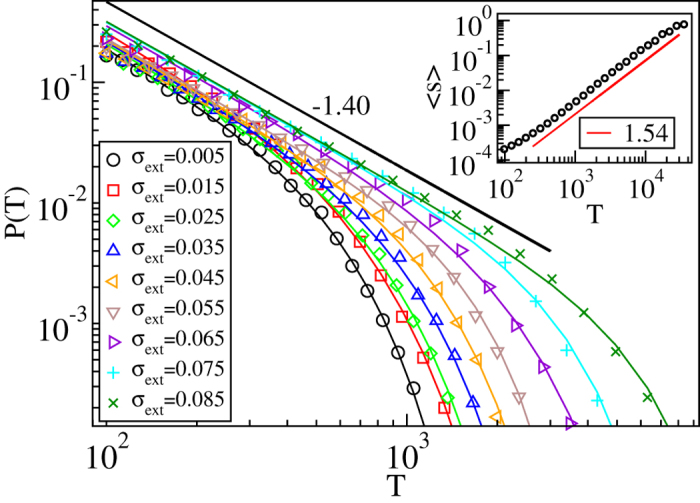
Criticality in durations of the deformation avalanches is in accordance with expectations from depinning phase transitions. Distributions of the avalanche durations *P*(*T*) for various stress bins below the critical stress *σ*_*c*_ ≈ 0.09 (with *N*_*s*_ and *A* as in Fig. 3), showing that *τ*_*T*_ ≈ 1.40. The solid lines correspond to fits of the form 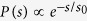. The inset shows the scaling of the average avalanche size 〈*s*〉 with the avalanche duration *T*, which follows 〈*s*〉 ∝ *T*^*γ*^, with *γ*  ≈ 1.54. Notice that both *τ*_*T*_ and *γ* have values that are clearly different from those of mean field depinning (i.e. 2 in both cases).

The results obtained in the limit of a small threshold value *V*_th_ = 10^−4^ in Fig. 3 show that the data is described by the scaling
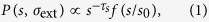
where *τ*_*s*_ = 1.30 ± 0.03 and *s*_0_ ∝ (*σ*_c_ − *σ*_ext_)^−1/*σ*^, with 1/*σ* = 1.90 ± 0.04. Notice that this behaviour, while in agreement with the standard depinning scaling picture, is fundamentally different from that observed in the corresponding pure system, where *s*_0_ is proportional to the exponential of the applied stress, *s*_0_ ∝ exp(*σ*_ext_/*σ*_0_), and the power law exponent *τ*_*s*_ has a lower value *τ* ≈ 1.0^19^. We have checked that *τ*_*s*_, as well as the cutoff of the distribution of slip *sL*^2^, are independent of the system size (see Supplementary Figs. S1 and S2). The latter result is again in contrast to the pure system results, where the slip distribution cutoff was found to exhibit a power law dependence on the number of dislocations, or the system size^19^. Our estimates of *τ*_*s*_ and 1/*σ* are close but not equal to their mean-field depinning values (3/2 and 2, respectively)^20^. The inset of the upper panel of Fig. 3 shows the stress-integrated distribution 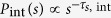, with *τ*_*s*,int_ = 1.85 ± 0.10. *τ*_*s*,int_ obeys within error bars the scaling relation *τ*_*s*,int_ = *τ*_*s*_ + *σ*^29^, and is also in reasonable agreement with the exponent value describing the distribution of dissipated energy during avalanches obtained from a minimal automaton model of 2*d* crystal plasticity^30^. It is worth noting that the range in which such critical scaling applies here (for the parameters *N*_s_ and *A* chosen in order to reduce any “transient time”, as seen in the simulations with constant external stress, and to ensure a significant difference wrt. the disorder-free system) is very wide in external stress (the control parameter), in agreement with experiments. We have checked the robustness of our results by considering three different values for *A*, all corresponding to the “pinning” phase in Fig. 1: all cases yield the same exponent characterising the avalanche size distributions (see Supplementary Fig. S3). Similar conclusions are reached when looking at the avalanche durations, *P*(*T*,*σ*_ext_). Again (Fig. 4), a wide scaling regime ensues. The data now indicates a 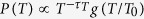 -scaling, with *τ*_*T*_ = 1.40 ± 0.05. The inset of Fig. 4 shows that the usual duration vs size -relation of crackling noise holds, in that 〈*s*(*T*)〉 ∝ *T*^*γ*^ with *γ* = 1.54 ± 0.05^31^. Both of these last exponents in particular, *τ*_*T*_ and *γ*, have values clearly different from their mean-field depinning counterparts (2 for both *τ*_*T*_ and *γ*).

**Figure 5:**
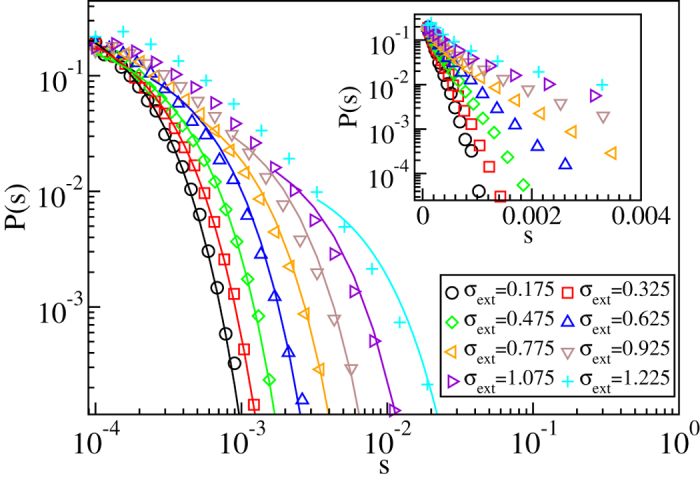
Very strong disorder quenches the critical behaviour. For *N*_s_ = 32000 and *A* = 1.0, the avalanche size distributions are no longer power laws. Instead, a pure exponential function 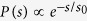 results in a better fit. The inset shows the same distributions as in the main figure, but with semi-logarithmic axes.

Above, we have shown that a depinning-like criticality can be established by fixing suitable, non-zero values for the disorder strength parameters *A* and *N*_*s*_. Obviously, by lowering *A*, one approaches the disorder-free case of dislocation jamming. We do not look at the interesting issue of how crossing the phase boundary looks like when moving from jamming to pinning (or vice versa). One would expect a kind of “Larkin length” to ensue, such that when a dislocation avalanche spans a large enough area to explore the random impurity landscape, it would show depinning-like characteristics instead of those related to jamming, consider again the insets of Fig. 2. It is a natural question to ask what happens if in the competition between long-range dislocation-dislocation interactions and the local effect of the pinning sites/solute atoms the latter starts to dominate. In Fig. 5, we show the outcome for *A* = 1.0, *N*_s_ = 32000, such that the forces experienced by the dislocations due to quenched pinning are much larger than those due to dislocation interactions. Now, all signs of power-law like avalanche activity are absent, and an exponential distribution of avalanche sizes is found, 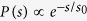, where 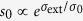, and *σ*_0_ ≈ 0.28. Note that as expected, given the change of *P*(*s*), the avalanche sizes are now much more limited than in Fig. 3, see also Supplementary Movies 1 and 2, showing examples of the intermediate and strong disorder cases, respectively. A similar avalanche size-limiting effect due to strong pinning has been shown for superconducting vortex avalanches^32^. This result confirms the third, strong-disorder phase stipulated in the phase diagram (Fig. 1).

## Discussion

In summary, we have studied the dynamics of 2*d* dislocation assemblies in the presence of disorder. The model has a phase diagram (see Fig. 1) that contains three phases. As a special case one has the usual disorderless one^19^, and then two where quenched disorder is important: A strong disorder one, where collective dynamics does not exist, and another one with critical behaviour typical of the depinning of elastic manifolds, and with a set of exponents different from the mean-field limit of this class of systems, despite the long-range nature of the dislocation interactions. Our results leave fundamental questions about the phase diagram presented. We have argued that the mixing of long-range interactions and disorder leads to two new phases, one in which dislocation interactions are partly screened leading to depinning-like criticality with non-trivial exponents, and another where critical behaviour is absent due to strong screening. The fundamental issues concern now the details of the phase diagram, the cross-overs from jamming to pinning and vice versa, and the precise location of the point where the jammed, pinned, and the flowing phases meet.

Which of the three phases is observed for a specific crystal should depend on the densities of dislocations and pinning centres, and on the pinning strength induced by the latter: the fluctuating dynamics of a mobile dislocation is controlled by the spatial fluctuations of the forces of different origins experienced by it as it moves, i.e. those originating from dislocation interactions (a dynamic quantity), and from interactions with the static pinning centres. For instance, decreasing the dislocation density in a system with a fixed concentration and strength of pinning centres will eventually lead to a situation where pinning forces experienced by the dislocations dominate over those due to dislocation interactions. Another consequence of adding a quenched pinning field is given by the introduction of a microscopic disorder length-scale to the otherwise scale-free dislocation system, implying that the disordered dislocation system is not in the “similitude regime”^33^: an extreme manifestation of this is given by the lack of scale invariance of the strain bursts for very strong disorder.

Our study has been confined to the 2*d* case for the basic reason that collecting the large avalanche statistics needed in 3*d* DDD simulations is numerically much more challenging. There, in the absence of disorder, mean-field -like exponents have been claimed - although stress-resolved avalanche distributions were not considered in^7^ - and it follows naturally from our results that one expects to find the two screened phases also there, with the introduction of point-like pinning centres. In 3*d* systems, further complications may arise due to forest hardening: immobile dislocations on inactive slip systems could have a similar effect as our quenched pinning centres, possibly leading to pinning-dominated dislocation dynamics even in the absence of additional impurities such as solute atoms or precipitates. In BCC metals, also sufficiently strong Peierls barriers may have a similar effect. Two-dimensional systems such as colloidal crystals^34^ may provide relevant experimental systems to directly test our results. Note that a similar set of exponents to the one observed here for the intermediate disorder strength case was found very recently in a 2*d* amorphous plasticity model^35^, suggesting a possibility of a broad universality class of plastic deformation, where microscopic details are irrelevant.

A most important practical conclusion is that the micro-structure of materials with dislocation activity may induce discrete qualitative changes in the bursty deformation dynamics: jamming or pinning. The depinning phase should give rise to usual phenomena such as thermally assisted creep^36^ and glassy relaxation^37^, which relate to the critical exponents of the transition, and where the spatial correlations (point- or line-like and so forth) of the disorder are relevant. An obvious further generalisation of our study is to time-dependent disorder, such as diffusing solute fields^38^, where phenomena such as the Portevin-Le Chatelier effect should ensue^39^,^40^.

## Methods

### 2D DDD model with pinning

The 2*d* DDD model we study is a development of other models studied in the literature^26^,^27^, with the addition of a random arrangement of *N*_s_ quenched pinning centres. It represents a cross section (*xy* plane) of a single crystal, with a single slip geometry, and straight parallel edge dislocations along the *z* axis. The *N*_d_ edge dislocations glide along directions parallel to their Burgers vectors *b* = ±*b***u**_*x*_, where *b* is the magnitude and **u**_*x*_ is the unit vector along the *x* axis. Equal numbers of dislocations with positive and negative Burgers vectors are assumed, and dislocation climb is not considered: The latter is a good approximation for low temperatures^41^,^42^. The dislocations interact with each other through their long-range stress fields, *σ*_d_(**r**) = *Dbx*(*x*^2^ − *y*^2^)/(*x*^2^ + *y*^2^)^2^, where *D* = *μ*/2*π*(1 − *ν*), with *μ* the shear modulus and *ν* the Poisson ratio of the material. In addition, we consider a random arrangement of *N*_s_ immobile solute atoms interacting with the dislocations via short-range interactions. To this end, we use the regularised interaction energy derived from non-local elasticity^43^, expressed in polar coordinates for the *n*th dislocation with a sign *s*_*n*_ = ±1 as
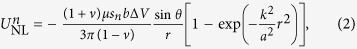
where Δ*V* is the misfit area, *k* = 1.65, and *a* is the atomic distance^43^. This regularised form of the interaction energy removes the singularity at *r* = 0. Other short-range pinning potentials should lead to similar results: we have checked that this is true for Gaussian pinning centres with 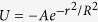 (see Supplementary Fig. S4). The corresponding interaction force acting on the dislocations due to a solute atom is then given by ****F****_ds_ = −∇*U*_NL_.

Thus, the overdamped equations of motion of the dislocations read
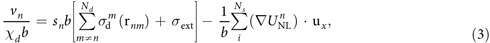
with *v*_*n*_ the velocity and *s*_*n*_ the sign of the nth dislocation, *χ*_d_ the dislocation mobility (implicitly including effects due to thermal fluctuations), and *σ*_ext_ is the externally applied stress. The dislocation-solute force decays with distance as 1/*r*^2^ (while the dislocation-dislocation force ~1/*r*), and we introduce a cut-off distance *r*_cutoff_ = 15*b* (corresponding typically to two times the average dislocation-dislocation distance) beyond which the dislocation-solute interaction is set to zero. At the cut-off distance, the dislocation-solute interaction is several orders of magnitude smaller than typical dislocation-dislocation interactions, and thus has a negligible effect on dislocation dynamics. The equations of motion are integrated with an adaptive step size fifth order Runge-Kutta algorithm, by measuring lengths in units of *b*, times in units of 1/(*χ*_d_*Db*), and stresses in units of *D*, and by imposing periodic boundary conditions in the *x* direction. Two dislocations of opposite sign, with a mutual distance smaller than *b*, are removed from the system, to include a mechanism for dislocation annihilation in the model.

The simulations are started from a random initial configuration of *N*_d_ = 1600 dislocations within a square cell of linear size *L* = 200*b*. These initial states are first relaxed with *σ*_ext_ = 0, to reach metastable dislocation arrangements; Fig. 1 shows a local detail of such a system. After the annihilations during the relaxation, *N*_d_ ≈ 800–900 dislocations remain. Then, an external stress is turned on, and the evolution of the system is monitored, by measuring the time dependence of various quantities such as the strain rate,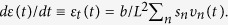 In the simulations, we consider the effect of varying both the dislocation-solute interaction strength *A* = (1 + *ν*)*μb*Δ*V*/3*π*(1 − *ν*) and the solute density *ρ*_*s*_ = *N*_s_/*L*^2^. In the absence of correlations, *Aρ*_*s*_ measures the relative strength of disorder, to be compared to the strength of the dislocation-dislocation interactions. The range of values considered for the disorder parameters is *A* = 0.05–1.0 and *N*_s_ = 500–32000, resulting in *Aρ*_*s*_ ≈ 10^–3^–1.0. The results are averaged over a large number of realisations for each set of parameters, ranging from 500 to 6000.

## Additional Information

**How to cite this article**: Ovaska, M. *et al.* Quenched pinning and collective dislocation dynamics. *Sci. Rep.* **5**, 10580; doi: 10.1038/srep10580 (2015).

## Supplementary Material

Supporting Information

Supporting Information

Supporting Information
