# Supplementary material for: Quenched pinning and collective dislocation dynamics
Source: Sci Rep. 2015 May 29;5:10580. doi: 10.1038/srep10580 (PMC4650641; doi:10.1038/srep10580)
Supplement: Supporting Information [file srep10580-s1.pdf]

Supplementary Information: Quenched pinning and collective dislocation dynamics

Markus Ovaska, Lasse Laurson, and Mikko J. Alava

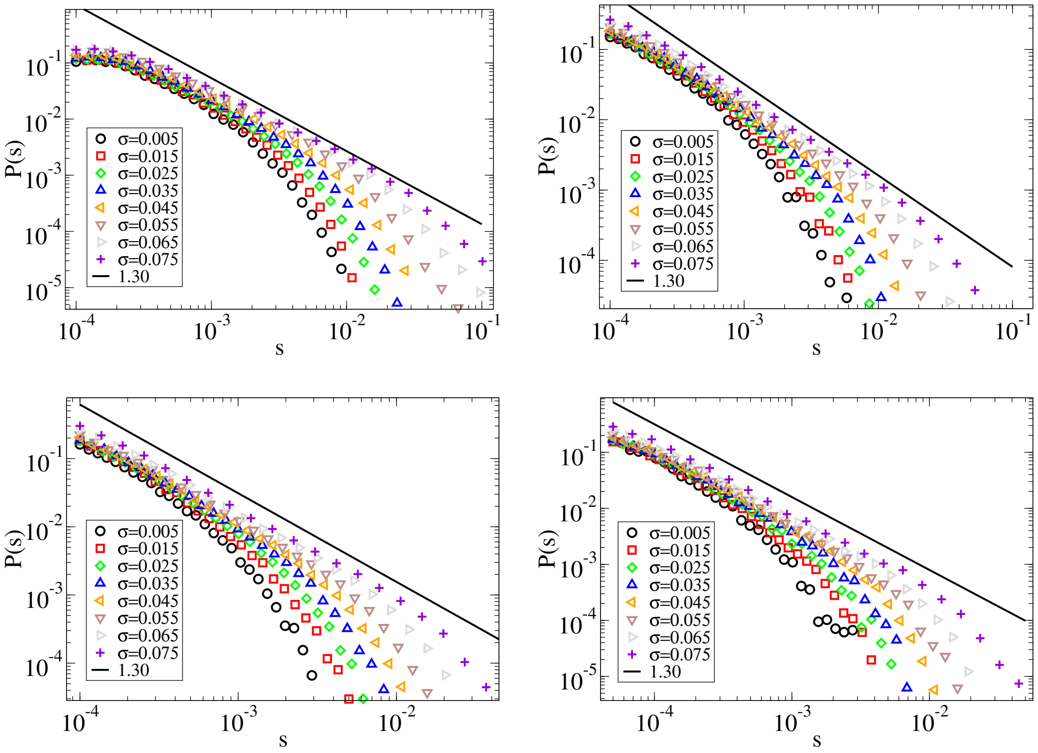

Supplementary Figure S1: Strain burst size distributions for different system sizes. The initial dislocation density is $\rho_d = N_d/L^2 = 0.04$, and the pinning center density is $\rho_s = N_s/L^2 = 0.8$ for all system sizes. **Top left:** $L = 100$. **Top right:** $L = 150$. **Bottom left:** $L = 200$. **Bottom right:** $L = 250$. Upon increasing the system size, the power law scaling regime characterized by the exponent $\tau_s = 1.3$ (black lines) becomes more extended. For system sizes $L \geq 150$ in particular, a very good fit with $\tau_s = 1.3$ is obtained, independent of L, highlighting the absence of finite-size effects masking the scaling behavior.

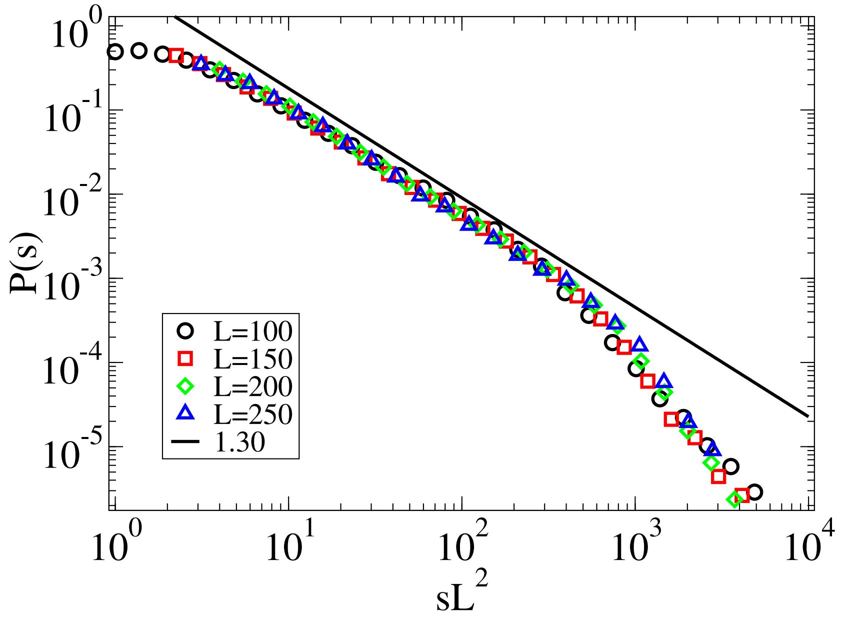

Supplementary Figure S2: Distributions of slip sL^2 produced by the strain avalanches for $\sigma_{ext} = 0.075$ for different system sizes L. The fact that the curves collapse indicates the absence of finite-size effects, and also that the usual depinning scaling where the avalanche cutoff is controlled by the external stress (rather than L as in the system without pinning) applies.

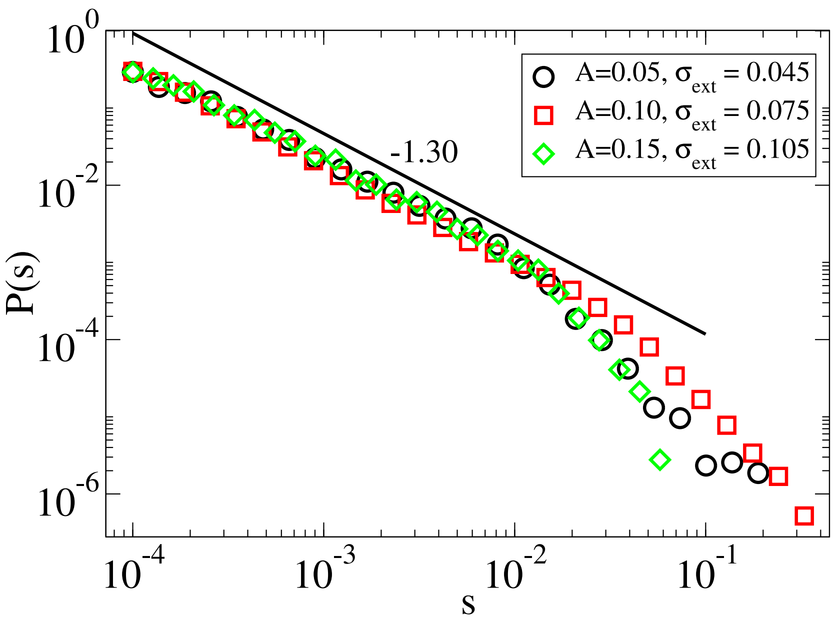

Supplementary Figure S3: Strain burst size distributions for different pinning strengths A, when the external stress is close to the the critical stress σ_c. The data shows that the exponent $\tau_s = 1.3$ characterizing avalanche size distributions in the pinning phase is robust wrt. small changes in pinning strength. The system size is $L = 200$ and the number of pinning centers is $N_s = 32000$.

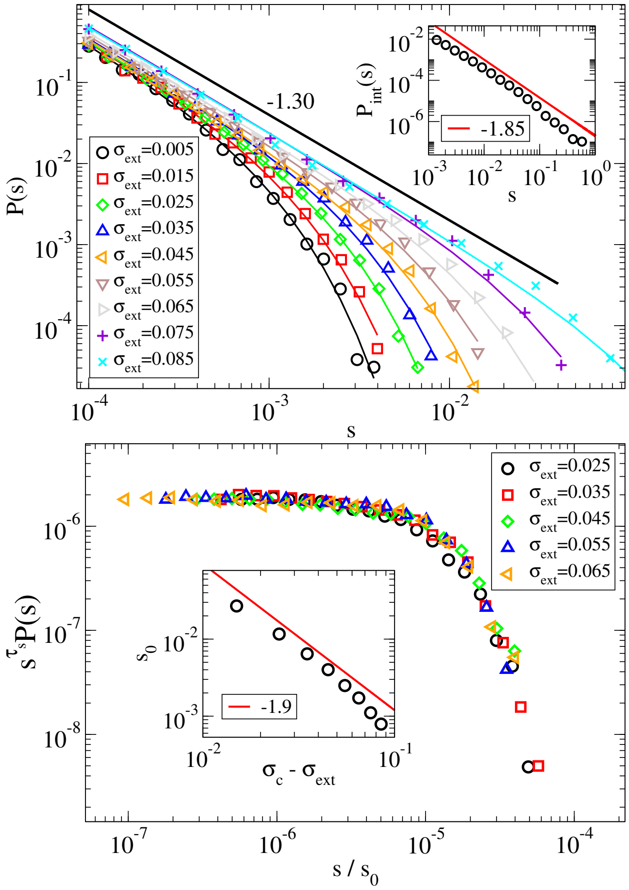

Supplementary Figure S4: Similar data as in Fig. 3 of the manuscript, but obtained by using Gaussian pinning centers, with the interaction potential given by $U = -Ae^{-r^2/R^2}$, with parameters $A = 0.05$ and $R = 1.0$, chosen to reproduce the pinning strength in Fig. 3 of the paper. The main figure of the top panel shows the avalanche size distributions for different stress levels σ_{ext}, while the inset shows the stress-integrated avalanche size distribution. The main figure of the bottom panel shows a collapse of the distributions, and the corresponding inset the scaling of the cut-off s_0 with the distance to the critical stress $\sigma_c - \sigma_{ext}$. The fitted exponents are the same as in Fig. 3 of the paper, i.e. $\tau_s = 1.3$, $1/\sigma = 1.9$, and $\tau_{s,int} = \tau_s + \sigma \approx 1.85$, illustrating the fact that any short range uncorrelated pinning field is expected to have the same effect on the statistics of the strain bursts.

Legends for Supplementary Movies 1 and 2:

Supplementary Movie 1 (Movie1.mov):

Dislocation dynamics with disorder of intermediate strength. An example of a single simulation run with an intermediate disorder strength, corresponding to the phase showing depinning-like criticality of the avalanche dynamics.

Supplementary Movie 2 (Movie2.mov):

Dislocation dynamics with very strong disorder. An example of a single simulation run with very strong disorder, corresponding to the phase where criticality is quenched by the strong disorder, resulting in exponential avalanche size distributions.
